# Interventions to Increase Colorectal Cancer Screening Uptake in Rural Settings: A Scoping Review

**DOI:** 10.5888/pcd22.250025

**Published:** 2025-07-17

**Authors:** Christine M. Kava, Judith Lee Smith, Emily K. Kobernik, Jan M. Eberth, Cynthia French, Sarah H. Nash, Whitney E. Zahnd, Ingrid J. Hall

**Affiliations:** 1 Division of Cancer Prevention and Control, National Center for Chronic Disease Prevention and Health Promotion, Centers for Disease Control and Prevention, Atlanta, Georgia; 2 Drexel University, Department of Health Management and Policy, Philadelphia, Pennsylvania; 3 University of Iowa, Department of Epidemiology, Iowa City; 4 University of Iowa, Department of Health Management and Policy, Iowa City

## Abstract

**Introduction:**

An estimated 6,000 preventable cancer deaths — including from colorectal cancer (CRC) — occurred in rural America in 2022. Screening can prevent CRC or identify disease at earlier stages when it is more treatable. However, national estimates for CRC screening lag behind Healthy People 2030 objectives. In rural settings, barriers to screening are unique and persistent.

**Methods:**

We performed a scoping review to describe the types and effectiveness of interventions to increase CRC screening in primarily rural settings. We included US-based studies published during January 2010 through May 2024. Interventions were categorized according to US Community Preventive Services Task Force–recommended strategies for multicomponent interventions.

**Results:**

Of 508 unique publications identified, 36 met inclusion criteria. Most studies were multicomponent interventions (n = 34). Most studies were associated with an increase in CRC screening uptake. The most common intervention approaches were client reminders (eg, telephone reminders about screening) (n = 25), small media (eg, pamphlets) (n = 25), and reducing structural barriers to screening (eg, patient navigation) (n = 24). Over half (n = 21) of studies reported using a theory, framework, or research approach to inform intervention development, implementation, or evaluation. Six studies (17%) included cost evaluations. The studies included in this review represented less than half of all US states.

**Conclusion:**

This scoping review provides insight into CRC screening intervention implementation in rural settings. The limited geographic representation of the interventions included in our review may highlight an opportunity to improve implementation and dissemination of effective CRC screening interventions in rural settings to reduce CRC incidence and death.

**Summary**

**What is already known on this topic?**

Screening can prevent colorectal cancer (CRC), but rural communities face screening barriers. Reviews have not examined implementation of interventions to increase CRC screening in primarily rural settings.

**What is added by this report?**

This scoping review examined implementation of CRC screening interventions in primarily rural settings. Most studies in our review implemented multicomponent interventions and were effective at increasing CRC screening uptake.

**What are the implications for public health practice?**

Intervention implementation may be improved by using theory-based approaches, assessing costs, and expanding populations covered. Several areas of the country were not included in identified studies in this report, which may suggest a need for expanded intervention implementation tailored to community need and context.

## Introduction

Rural populations in the US have higher rates of colorectal cancer (CRC) incidence and deaths compared with urban populations (1–3). Use of screening tests as recommended by the US Preventive Services Task Force (USPSTF) (4) and the American College of Gastroenterology (5) can help detect and prompt treatment to remove most precancerous polyps before they develop into cancer (6). Screening can also reduce the risk of death (7) by finding tumors early — when treatment is most effective. However, the current prevalence of CRC screening (63.5%) nationally lags behind the prevalence of breast (79.8%) and cervical (73.9%) cancer screening and the recommended Healthy People 2030 target of 72.8% (8).

People living in rural versus urban settings are less likely to receive CRC screening (9,10). This screening disparity may partially explain the association of rural residency with presentation of advanced disease (11). However, even when controlling for screening, recent estimates suggest that rural US counties have higher rates of CRC deaths (12). People in some racial and ethnic minority groups in rural settings are less likely to be screened compared to their urban counterparts (13). The disparity between urban and rural populations may be due to limited access to quality health care and delays in obtaining timely, recommended treatment — given longer travel distances, transportation difficulties, provider shortages, and limited access to specialty care (14).

Previous reviews of CRC screening have included studies conducted in rural settings in combination with other populations regardless of rurality (eg, rural and low-income populations [10]). To our knowledge, no previous reviews have focused on CRC screening interventions in primarily rural settings only. We performed a scoping review to describe interventions implemented in rural settings to increase CRC screening and identify how effectiveness might differ across populations. Included studies were categorized according to the Community Preventive Services Task Force (CPSTF)-recommended intervention strategies: increase community demand, increase community access, and increase provider delivery of screening services (15).

## Methods

We performed a scoping review in May and June 2024. The general purpose of a scoping review is to identify and map available evidence on a given topic (16). We identified available evidence on CRC screening interventions conducted in primarily rural settings. We followed Preferred Reporting Items for Systematic Reviews and Meta-Analyses extension for Scoping Reviews (PRISMA-ScR) guidelines (17). Like previous scoping reviews (18,19) and aligned with the defining characteristics and reporting requirements for a scoping review (16,17), our assessment did not report on study quality or risk of bias.

### Data sources

We developed a search strategy in consultation with a librarian. Search engines were CINAHL (Cumulative Index to Nursing and Allied Health Literature), Embase, Medline, and Scopus. Example search terms included colorectal, neoplasms, screen, colonoscopy, FOBT (fecal occult blood test), immunochem, sigmoidoscopy, stool, rural, intervention, and program (the Appendix includes the full search strategy and terms). Eligibility criteria for our search included English-language publications in peer-reviewed journals during January 2010 through May 2024. We did not limit our search to specific CRC screening tests or study designs, because we wanted to capture data on a wide range of CRC screening interventions. We identified 1,107 records from our search. After removing duplicates (n = 599), there were 508 records for abstract review based on our inclusion and exclusion criteria.

### Study selection

**Figure 1:**
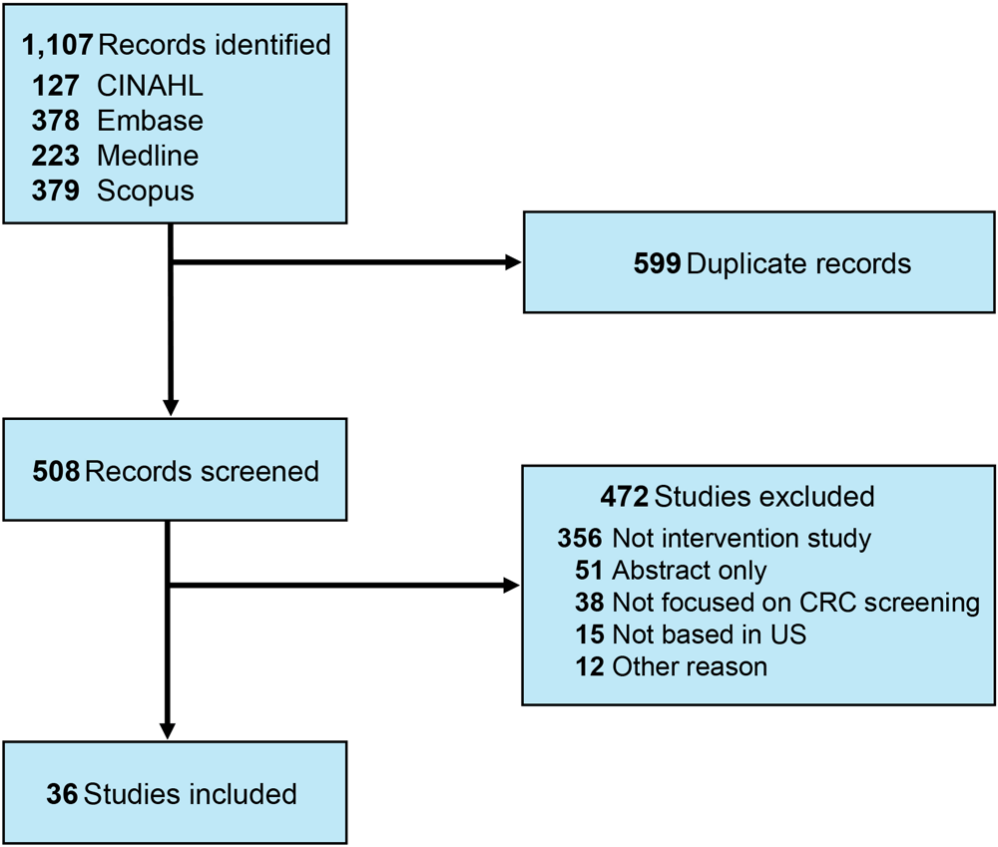
Article search and selection, scoping review of colorectal cancer (CRC) screening interventions in rural settings, January 2010–May 2024. “Other reason” for excluding research from this review were that, eg, the item was a book chapter or that the research was not focused on rural settings. Abbreviation: CINAHL, Cumulative Index to Nursing and Allied Health Literature.

Abstracts were divided among 4 members of the research team (C.M.K., E.K.K., J.L.S., I.J.H.) for review. Given country-level differences in cancer screening capacity, coverage, and guidelines, only studies conducted in the US were included. We included studies that reported being focused primarily on rural settings. We excluded studies focused on urban settings, even if they serviced rural populations. Studies that did not implement or evaluate an intervention for CRC screening were excluded. We also excluded conference abstracts and book chapters. Of the 508 abstracts reviewed, 36 studies met our inclusion criteria (Figure 1).

### Data extraction

We abstracted data from the 36 included studies into an Excel (Microsoft) spreadsheet and summarized the following: study design, population, and setting; whether the authors provided a formal definition of rurality; screening tests and guidelines used; intervention description; sample size(s) and characteristics; and summary of results. We categorized interventions according to the 3 CPSTF-recommended strategies for multicomponent interventions and used the abstracted data to develop summary tables and findings.

## Results

### Study characteristics

**Figure 2:**
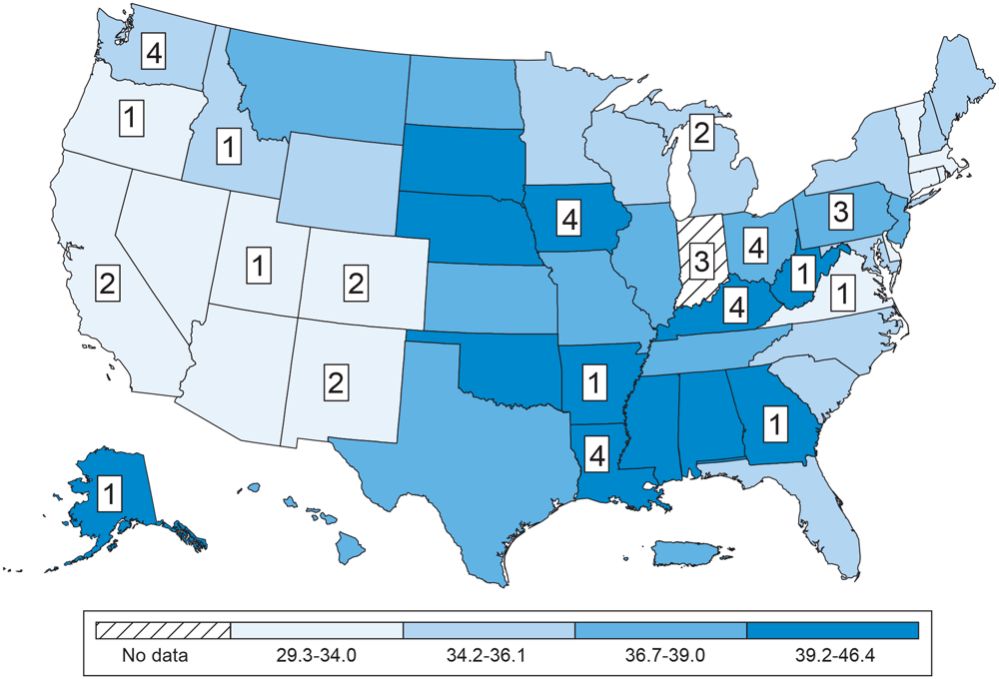
Age-adjusted colorectal cancer incidence and distribution of included articles (N = 36) by state, scoping review of colorectal cancer screening interventions in rural settings, January 2010–May 2024. Age-adjusted colorectal cancer incidence rates per 100,000 standard population were based on US cancer incidence data from 2017 through 2021. The numbers represent the number of articles that were included in our review whose intervention was implemented in that state. Four pairs of articles (n = 8 total) were publications on the same study.

The 36 articles included in our review evaluated populations from 19 states (Table 1, Figure 2). Four pairs of articles (n = 8 total) described the same study. The states with the most articles published were Iowa, Kentucky, Louisiana, Ohio, and Washington (n = 4, each). Studies were primarily conducted in health care settings, including federally qualified health centers (FQHCs), tribally operated health care facilities, and other community health clinics and systems.

**Table 1:** Characteristics of Included Studies (N = 36), Scoping Review of Colorectal Cancer (CRC) Screening Interventions in Rural Settings, January 2010–May 2024

Study	Location	Population	Study setting	Rural defined^b^	Sample^a^			
					N	Mean age, y	Sex	Race and ethnicity
Adegboyega et al 2022 (20)	Kentucky	Appalachian residents aged ≥50 years	Large rural medical center	No	190	57.8	59% Women; 41% men	98% White; 2% another identity
Arnold et al 2016 (21)^c^	Louisiana	Patients aged 50–85 years	3 FQHCs	No	961	58.4	77% Women	67% African American; 33% Caucasian/Hispanic
Arnold et al 2019 (22)^d^	Southern Louisiana	Patients aged 50–75 years	4 Rural community clinics	No	614	58.4	55% Women	67% African American; 33% Hispanic or White
Briant et al 2015 (23)	Washington State	People aged ≥18 years	3 Rural counties	No	947	NR	77% Women; 23% men	76% Hispanic; 12% Non-Hispanic White
Briant et al 2018 (24)	Eastern Washington State	People aged ≥50 years	Rural Hispanic community	No	101	NR	69% Women; 30% men	99% Hispanic; 1% White
Charlton et al 2014 (25)	Iowa City, Iowa	Veterans aged 51–64 years	Catchment area of large rural population	Yes	1,499	58.7–60.1	84%–99% Men	95% White^e^
Christiansen et al 2016 (26)	Northern California	Established patients	3 Rural FQHCs	No	6,023	NR	54% Women; 46% men	NR
Conn et al 2020 (27)	West Virginia	Patients	9 FQHCs	No	NR	NR	NR	NR
Curry et al 2011 (28)	Pennsylvania	Medical record review of patients aged ≥50 years	4 Primary care practices	No	301–323	NR	NR	NR
Davis et al 2013 (29)^c^	Northern Louisiana	Patients aged 50–85 years	3 FQHCs	Yes	961	58.4	77% Women	67% African American; 33% Caucasian/Hispanic
Davis et al 2020 (30)^d^	Louisiana	Patients aged 50–75 years	4 Rural community clinics	Yes	568	58.4	55% Women	67% African American; 33% Caucasian/Hispanic
Davis et al 2023 (31)	Oregon	Health care plan enrollees aged 50–75 years	3 Health clinics	Yes	169	NR	59% Women; 40% men	8% Hispanic/Latinx; 84% Non-Hispanic /Latinx; 8% unknown
Dignan et al 2014 (32)	Kentucky	Medical record review of patients aged ≥50 years	66 Primary care practices	No	3,751–3,844	64.1–64.8	60%–61%	NR
Hardin et al 2020 (33)	Hazard, Kentucky	Patients aged 50–74 years	FQHC in Appalachia	No	NR	NR	NR	NR
Haverkamp et al 2020 (34)	New Mexico	American Indian and Alaska Native patients aged 50–75 years	3 Tribally operated health care facilities	No	1,288	60.4	52% Women	NR
Hirko et al 2020 (35)	Michigan	Patients aged 50–75 years	Largely rural health system	Yes	7,812	NR	54% Women; 46% men	NR
Honeycutt et al 2013 (36)	Southwest Georgia	Patients aged 50–64 years with low income or underinsured or uninsured	13 Community health clinics	No	809	55.8	67% Women; 33% men	63% Black; 37% White
Hountz et al 2017 (37)	Indiana	Medical record review of patients aged 51–74 years	Nurse–managed health clinic	No	400	NR	58%–68% Women; 32%–42% men	88%–96% Caucasian; 4%–10% Hispanic; 1%–2% another identity
Katz et al 2024 (38)	Scioto County, Ohio	Patients aged 50–64 years	FQHC	Yes	94	57.4	50% Women	NR
Kinney et al 2014 (39)	California, Colorado, Idaho, New Mexico, Utah	Relatives of CRC patients aged 30–74 years at high risk	State cancer registries	Yes	481	50.3	57% Women; 43% men	4% Latino; 94% Non-Latino White; 2% another identity
Klugas et al 2024 (40)	Michigan	Patients aged 50–75 years	Rural family medicine clinic	No	407	63.3	54% Women; 45% men	0% Black/African American; 2% Hispanic; 98% White; 0.7% another identity
Kluhsman et al 2012 (41)	Pennsylvania	Patients aged ≥50 years	3 Rural primary care practices	No	200	61.0	75% Women; 25% men	NR
Krok–Schoen et al 2015 (42)	Ohio	People aged 51–75 years	Selected from InfoUSA County Directories	No	4,491	61.2–62.6	55%–60% Women; 40%–45% men	94%–97% White; 0.0%–2% Hispanic^f^
Levy et al 2012 (43)^g^	Iowa	Patients aged 52–79 years	16 Rural family physician offices	No	373	61.2	52% Women; 48% men	0.3% Asian; 0.5% Black; 99% White; 0.8% Hispanic^f^
Levy et al 2013 (44)^g^	Iowa	Patients aged 52–79 years	16 Rural family physician offices	No	743	NR	96%–99% Women	0%–0.5% Asian; 0%–1% Black; 98%–99.5% White; 0%–2% another identity; 0.5%–2% Hispanic^f^
Moralez et al 2012 (45)	Eastern Washington State	People aged 50–79 years	Rural Hispanic community	No	61^h^	57.9	72% Women	100% Hispanic
Moss et al 2024 (46)	Pennsylvania	Female patients aged 50–65 years	9 FQHCs in racially segregated counties	Yes	48	55.8	100% Women	83% White; 17% another identity
Preston et al 2018 (47)	Mississippi County and St. Francis County, Arkansas	County residents	2 underserved, low-income counties	No	330	NR	NR	70% African American; 14% White; 1% another identity; 15% did not report
Rawl et al 2023 (48)^i^	Indiana, Ohio	Female residents aged 50–74 years	98 Rural counties	No	542	58.8	100% Women	98% White; 3% another identity
Redwood et al 2012 (49)	Alaska	Alaska Native residents	Rural and remote Alaska	No	518	NR	52% Women; 48% men	100% Alaska Native
Schlauderaff et al 2017 (50)	Mason County, Washington	Patients aged 50–75 years	Rural health clinic	No	1,208	NR	NR	NR
Schlichting et al 2014 (51)	Iowa City, Iowa	Veteran patients aged 51–60 years	Veterans Affairs health care system	No	473	60.0	87%–96% Men	92%–95% White
Vachon et al 2024 (52)^i^	Indiana, Ohio	Female residents aged 50–74 years	98 Rural counties	Yes	663	58.0	100% Women	98% White; 2% another identity
Westfall et al 2013 (53)	Eastern Colorado	Local residents aged ≥50 years	16 Eastern counties	No	1,050	NR	67% Women; 33% men	1%–2% American Indian or Alaska Native; 88%–90% White; 7%–8% another identity^j^; 2% don’t know/declined race; 7%–10% Hispanic^f^
Woodall and DeLetter 2018 (54)	Hopkins County, Kentucky	Employees aged ≥40 years	Local city government and community hospital	No	186	40.6	8% Women; 91% men	1% American Indian or Alaska Native; 9% Black/African American; 90% White; 1% another identity; 1% missing
Zoellner et al 2023 (55)	Southwestern Virginia	Age-eligible patients who received FIT orders during visit	Rural 3-clinic FQHC	No	119	NR	65% Women	NR

Abbreviations: FIT, fecal immunochemical test; FOBT, fecal occult blood test; FQHC, federally qualified health center; NR, not reported.

a Sample characteristics are listed as reported. “Other” and “Non-White” responses are reported here as “another identity.” Some studies provided sample characteristics only by group (eg, preintervention vs postintervention) rather than in total; for these studies, the range of values across groups is reported. Percentages may not total 100% because of rounding or missing data.

b Whether the study provided a definition of rurality based on population size or established census-tract or county-level standards (eg, Rural–Urban Continuum Codes).

c Arnold et al 2016 and Davis et al 2013 report on the same intervention. Davis et al 2013 report on return of initial FOBT within 3 months and 12 months (initial results), while Arnold et al 2016 report return of 1, 2, or 3 FOBTs over a 3-year period (final results).

d Arnold et al 2019 and Davis et al 2020 report on the same intervention. Arnold et al 2019 report on return of initial FIT within 12 months, while Davis et al 2020 report on return of repeat FIT within 12 to 18 months.

e Race and ethnicity was asked only among eligible respondents who completed the survey, not among all respondents assigned to a study group.

f Ethnicity reported separately from race.

g Levy et al 2012 and Levy et al 2013 report on the same intervention. Levy et al 2012 report results for all 4 study groups (chart reminder group, mailed education group, mailed education and telephone reminder group, usual care control group), while Levy et al 2013 compare results between 2 study groups (mailed education group vs mailed education and telephone reminder group).

h Number of participants who participated in a CRC home health party and completed baseline and follow-up surveys; demographic information reported is based on this number. The total number of community residents who attended at least 1 CRC home health party was 252.

i Rawl et al 2023 and Vachon et al 2024 report results from the same intervention. Rawl et al 2023 report data on receipt, uptake, and satisfaction of the intervention, while Vachon et al 2024 report on CRC screening outcomes.

j Includes African American, Asian, Native Hawaiian or other Pacific Islander, and “other” responses. Refer to the article for detailed breakdown by race and ethnicity.

**Table:** 

State	Incidence range	Number of Articles
Alabama	39.2–46.4	0
Alaska	39.2–46.4	1
Arizona	29.3–34.0	0
Arkansas	39.2–46.4	1
California	29.3–34.0	2
Colorado	29.3–34.0	2
Connecticut	29.3–34.0	0
Delaware	29.3–34.0	0
District of Columbia	34.2–36.1	0
Florida	34.2–36.1	0
Georgia	39.2–46.4	1
Hawaii	36.7–39.0	0
Idaho	34.2–36.1	1
Illinois	36.7–39.0	0
Indiana	Not reported	3
Iowa	39.2–46.4	4
Kansas	36.7–39.0	0
Kentucky	39.2–46.4	4
Louisiana	39.2–46.4	4
Maine	34.2–36.1	0
Maryland	34.2–36.1	0
Massachusetts	29.3–34.0	0
Michigan	34.2–36.1	2
Minnesota	34.2–36.1	0
Mississippi	39.2–46.4	0
Missouri	36.7–39.0	0
Montana	36.7–39.0	0
Nebraska	39.2–46.4	0
Nevada	29.3–34.0	0
New Hampshire	34.2–36.1	0
New Jersey	36.7–39.0	0
New Mexico	29.3–34.0	2
New York	34.2–36.1	0
North Carolina	34.2–36.1	0
North Dakota	36.7–39.0	0
Ohio	36.7–39.0	4
Oklahoma	39.2–46.4	0
Oregon	29.3–34.0	1
Pennsylvania	36.7–39.0	3
Rhode Island	29.3–34.0	0
South Carolina	34.2–36.1	0
South Dakota	39.2–46.4	0
Tennessee	36.7–39.0	0
Texas	36.7–39.0	0
Utah	29.3–34.0	1
Vermont	29.3–34.0	0
Virginia	29.3–34.0	1
Washington	34.2–36.1	4
West Virginia	39.2–46.4	1
Wisconsin	34.2–36.1	0
Wyoming	34.2–36.1	0

Nine studies defined rurality (Table 1). The underlying measures used to define rurality varied across studies and included Rural–Urban Continuum Codes (56) (n = 3), rural–urban commuting area codes (57) (n = 4), and a description of population size (n = 2). With the exception of 1 study in which the mean age of the participants was less than 50 years old (54), the mean age of study participants ranged from 50.3 to 64.8 years among the 21 studies that reported this information. Most participants in most studies were female and White.

Twenty-one studies reported using a theory, framework, or research approach to inform intervention development, implementation, or evaluation (Table 2). Fifteen studies used a randomized controlled trial study design; other designs included pretest–posttest, quasi-experimental, and mixed methods. Most studies evaluated CRC screening uptake as a primary outcome. The screening tests evaluated were colonoscopy (n = 26), fecal immunochemical test (FIT) (n = 21), FOBT (n = 19), sigmoidoscopy (n = 12), barium enema (n = 5), and Cologuard (n = 2). Of studies that evaluated FIT or FOBT, 13 reported on positive test results. Of studies that evaluated colonoscopy, 7 provided data on colonoscopies as a diagnostic test after receipt of a positive FIT or FOBT result.

**Table 2:** Intervention Characteristics and Outcomes of Included Studies (N = 36), Scoping Review of Colorectal Cancer (CRC) Screening Interventions In Rural Settings, January 2010–May 2024

Study; screening tests^a^	Theories, frameworks, approaches^b^; study design	Primary CRC outcomes	Intervention description	Key findings^c^	Other results^c^
**Adegboyega et al 2022 (** 20 **)**					
Any test (colonoscopy, FOBT, or sigmoidoscopy)	Health Belief Model; pilot randomized controlled trial	CRC screening completion postintervention	**Intervention group**: Lay health advisors provided brief motivational interviewing session on CRC screening. **Control group**: Patients received brochures on cancer screening.	No significant differences in CRC screening completion between intervention group vs control group (12% vs 15%; *P* = .72).	The intervention vs control group reported higher susceptibility to CRC (*P* = .04). Participants who were older and reported financial inadequacy reported more CRC screening barriers.
**Arnold et al 2016 (** 21 **)^d^**					
Colonoscopy, FOBT	Health Belief Model, Social Cognitive Theory; quasi-experimental	Completion of 3 annual FOBT tests	**Enhanced usual care comparison group**: Recommendation to speak with provider about and complete CRC screening and receipt of FOBT kit. **Education intervention group**: Same as usual care group plus simplified FOBT instructions. Education intervention included illustrated pamphlet, short video, and teach-back techniques. **Nurse support intervention group**: Same as education group plus nurse-led motivational interviewing sessions and additional support.	Significant differences in completion of 3 annual FOBT tests between enhanced usual care group (4.7%), education group (11.4%), and nurse support group (13.6%) (*P* = .005). Of all enrollees, 10.4% completed 3 FOBTs, 16.9% completed 2 FOBTs, 33.1% completed 1 FOBT, and 39.6% did not complete an FOBT.	Of participants who completed FOBT within 3 months, 51 (10.0%) had a positive test result. Of eligible participants who completed repeat FOBT in year 2, four (1.9%) had a positive test result. Of eligible participants who completed repeat FOBT in year 3, 7 (7.0%) had a positive test result.
**Arnold et al 2019 (** 22 **)^e^**					
FIT	Health Belief Model, Social Cognitive Theory; randomized controlled trial	Return of initial FIT kit within 12 months	**Both intervention groups**: Brief literacy-informed education intervention. Receipt of FIT kit with step-by-step instructions using teach-back techniques. **Personal call intervention group**: Call from prevention counselor as reminder to complete FIT and discuss barriers to screening. **Automated call intervention group**: Automated calls as reminders to complete FIT; calls included motivational messages.	FIT return rates were comparable between automated call group (69.2%) and personal call group (67.0%) (*P* = .57).	No significant difference in return rates between study groups among participants with limited health literacy (*P* = .12) or adequate health literacy (*P* = .43). The positive FIT rate in both groups was 7%.
**Briant et al 2015 (** 23 **)**					
FOBT	Community-based participatory research; preintervention–postintervention	CRC screening familiarity and intent, FOBT return rate	Held 47 community health fairs with inflatable colon to provide education and awareness of CRC screening. Distribution of 300 FOBT kits.	Of 300 FOBT kits distributed, 226 (75.3%) were returned. Significant increases in familiarity of CRC screening by age, sex, and race and ethnicity. In fully adjusted model, aged ≥50 years (OR, 1.99; 95% CI, 1.20–3.32); being Hispanic (OR, 4.25; 95% CI, 2.47–7.32), and having a regular physician (OR, 1.96; 95% CI, 1.03–3.74) was associated with higher odds of CRC screening intent posttest.	NA
**Briant et al 2018 (** 24 **)**					
Colonoscopy, FOBT, sigmoidoscopy	Community-based participatory research; preintervention–postintervention	CRC screening-related awareness, knowledge, and behavior	Community members hosted *promotor(a)*-led “home health parties”; participants were taught about CRC screening via interactive presentations and discussion. Hosts were recruited from the community at outreach events. Eligible participants received free FOBT and resources on follow-up screening.	Significant increase in CRC screening awareness and knowledge (*P* values <.001). No significant change in behavioral intentions to obtain CRC screening (*P* = .08). FOBT screening rates significantly increased from 52%^f^ to 80% (*P* < .001). Endoscopy screening rates significantly decreased from 35% to 13% (*P* < .001).	NA
**Charlton et al 2014 (** 25 **)**					
Colonoscopy, FIT, gFOBT	None reported; randomized controlled trial	Overall CRC screening rate 6 months postintervention	**Education only intervention group**: Mailed educational materials on CRC screening. **Education plus FIT intervention group**: Same as education group plus FIT kit. **Usual care comparison group**: No intervention.	Among the overall study population, the education-plus-FIT group had significantly higher CRC screening (21%) vs the education only (6%) and usual care groups (6%) (*P* values <.001). Results were similar when restricting analysis to participants eligible to complete FIT.	No significant differences in CRC screening rates by rurality or sex. Among rural participants only, CRC screening rates were 23% in the education-plus-FIT group, 5% in the education-only group, and 5% in the usual care group. Of 64 participants who completed the FIT, 8 (12%) had a positive test result.
**Christiansen et al 2016 (** 26 **)**					
Any test (barium enema, colonoscopy, FOBT, or sigmoidoscopy)	None reported; quasi-experimental	CRC screening completion	Patients empaneled to a designated primary care provider. Other systems-level and practice-level changes included the formation of care teams, expansion and revision of care team roles, and creation of dashboards to share and promote clinical and productivity data.	CRC screening increased from 9% at baseline to 12% at 6 months and 19% at 12 months postempanelment.	Total project costs were $52,987. Average patient cycle time decreased by 12 min per patient, resulting in additional patients seen and an average of $2,212 per day in increased revenue.
**Conn et al 2020 (** 27 **)**					
FIT	Transtheoretical Model (Stages of Change); preintervention–postintervention	FIT return rate	Patients that did not return FIT kit within determined time frame received reminder telephone calls or letter from staff.	Reminder interventions achieved a 41.2% average return rate, resulting in an overall average return rate of 60.7% and 19.6 percentage-point increase in returns (*P* < .001). Increases in average return rates were significant for all but 1 health system.	Identifying and tracking patients ranged in costs from $515.52 to $18,043.20. Total costs of reminder telephone calls ranged from $131.40 to $1,972.08; for mailed reminders, $130.34 to $3,022.50. Overall average total cost of tracking and mailings per FIT kit returned was $60.18; average cost of only telephone and letter reminders per FIT kit returned was $11.20. In total, 539 patients had positive FIT result, with rates ranging from 7.1% to 32.1%.
**Curry et al 2011 (** 28 **)**					
Colonoscopy, FOBT, sigmoidoscopy	PRECEDE-PROCEED model (Predisposing, Reinforcing, and Enabling Constructs in Educational Diagnosis and Evaluation — Policy, Regulatory, and Organizational Constructs in Educational and Environmental Development); preintervention–postintervention	Patients being current with CRC screening recommendations, having received a CRC screening within past year	Three academic detailing (offering continuing medical education credit [nonmonetary incentive]) visits with providers and staff. Visits reinforced standard medical practices and covered information on screening, reimbursement and referral, counseling, and follow-up. Providers and staff provided with educational tools and information on county-specific cancer incidence and mortality.	Based on review of randomly selected medical records, 56% of patients were current on CRC screening preintervention vs 60% postintervention (*P* = .29). Being screened for CRC in the past year significantly increased from 17% to 35% (*P* < .001).	NA
**Davis et al 2013 (** 29 **)^d^**					
FOBT	Community-based participatory research, Health Belief Model, Social Cognitive Theory; quasi-experimental pilot	FOBT completion at 3 months postenrollment	**Education intervention group**: Same as usual care group plus simplified FOBT instructions. Education intervention included illustrated pamphlet, short video, and teach-back techniques. **Nurse support intervention group**: Same as education group plus nurse-led motivational interviewing sessions and additional support. **Enhanced usual care comparison group**: Recommendation to speak with provider about and complete CRC screening and receipt of FOBT kit.	FOBT completion was 38.6% in the enhanced usual care group, 51.7% in educational group, and 60.6% in education and nurse support group (*P* = .012). Patients in the nurse support vs enhanced usual care groups were more likely to be screened (*P* = .02). No significant differences in likelihood of screening between nurse support vs education group (*P* = .09) or education vs enhanced usual care group (*P* = .20).	Significant differences in screening completion across study groups among patients with limited literacy (*P* = .006) but not adequate literacy (*P* = .06). Incremental costs over usual care were $250 per additional person screened for the education intervention and $1,337 for the nurse support intervention.
**Davis et al 2020 (** 30 **)^e^**					
FIT	Health Belief Model, Social Cognitive Theory; randomized controlled trial	Completion of repeat FIT (return of FIT kit in years 1 and 2 of the study)	**Both intervention groups**: Brief literacy-informed education intervention. Receipt of FIT kit with step-by-step instructions using teach-back techniques. **Personal call intervention group**: Call from prevention counselor as reminder to complete FIT and discuss barriers to screening. **Automated call intervention group**: Automated calls as reminders to complete FIT. Mailed letter as encouragement to complete FIT in year 2 of the study alongside materials provided at enrollment.	Repeat FIT return rates were comparable in the personal call group (33.6%) and automated call group (36.5%) (*P* = .45).	Screening rates were higher among patients with adequate vs limited health literacy (*P* = .03). No significant difference in return rates between study groups among participants with limited health literacy (*P* = .63) or adequate health literacy (*P* = .77). Screening rates were higher among patients aged ≥60 years vs those aged 50 to 59 years (*P* = .04). No significant differences in screening rates found by race and ethnicity. Significant interaction effect between age and study group; the personal call vs automated call intervention was more effective among patients aged ≥60 years, while the automated vs personal call was more effective among patients aged 50 to 59 years (*P* = .01). In year 1, 44 (7.2%) had positive FIT result. In year 2, 18 (9%) had a positive FIT result.
**Davis et al 2023 (** 31 **)**					
Cologuard, colonoscopy, FIT	None reported; mixed methods	Return of mailed FIT kit and completion of any CRC screening at 12 months postmailing	Vendor-delivered automated telephone calls, receipt of mailed FIT kit, and clinic-initiated follow-up reminder calls.	Preliminary results showed that, of enrollees on deployment list, 21% completed a mailed FIT and 15% completed a clinic-distributed FIT or other screening. Of enrollees who completed screening, 58% completed mailed FIT, 23% clinic-distributed FIT, 16% colonoscopy, and 3% Cologuard. There was high program feasibility and acceptability, supported by perceived positive benefits to program, program ease and alignment with existing workflows, adequate staffing capacity, and practice facilitation.	NA
**Dignan et al 2014 (** 32 **)**					
Barium enema, colonoscopy, FOBT, sigmoidoscopy	None reported; randomized controlled trial	Documentation of CRC screening at 6-month follow-up	**Early intervention group**: Delivery of academic detailing (offering continuing medical education credit [nonmonetary incentive]) for primary health care providers. Included presentations on screening efficacy, clinical performance, patient counseling, and screening-friendly practice environment. **Delayed control group**: Received intervention after 6-month follow-up period.	No significant changes in CRC screening rates between early intervention group and delayed control group at 6-month follow-up. When restricting analysis to patients who received provider recommendation for CRC screening, there were significant increases in documented colonoscopy in early intervention group (15.7% increase) vs delayed control group (2.4% increase) (*P* < .01). No significant differences for FOBT (*P* = .82) or any screening (*P* = .06) observed.	NA
**Hardin et al 2020 (** 33 **)**					
Colonoscopy, FIT	None reported; preintervention–postintervention	FIT kit return rate and additional number of individual screens from usual care to implementation period.	Receipt of FIT kit and $10 gift card upon return of completed kit. Nurse navigator intervention, including discussion of screening options with patient, telephone or mailed reminders to complete screening, and assistance with screening barriers (eg, arranging transportation to clinic).	FIT return rate increased from 21.7% during the usual care period to 47.6% (*P* = .001) during the implementation period. Estimated 91 additional patients screened based on 353 FITs distributed during implementation.	Total intervention costs were $11,632.54, most of which came from patient navigation activities ($9,163.54). Estimated incremental cost per additional screen was $127.83.^g^ The percentage of positive FIT results was 12.5% during usual care period and 9.5% during implementation period.
**Haverkamp et al 2020 (** 34 **)**					
Colonoscopy, FIT	None reported; randomized controlled trial	Return of completed FIT kits	**Mailing alone intervention group**: Mailed FIT kits, instructions, and notification letter. **Mailing plus outreach intervention group**: Same as mailing alone group plus telephone or home visit follow-up from American Indian Community Health Representative if test not returned. **Usual care control group**: Patients received FIT only if provider recommended.	Percentage of completed FIT kits was 6.4% in usual care group, 16.9% in mailing alone group, and 18.8% in mailing plus outreach group. Significant differences in FIT completion between usual care group vs both intervention groups (*P* values <.01); no significant difference between intervention groups (*P* = .44).	Proportion of returned FIT kits increased with age (*P* = .02). No significant differences in FIT completion by sex (*P* = .52). Two rounds of Community Health Representative outreach resulted in an additional 7.5% of FIT kits returned in mailing plus outreach group. Of participants who returned FIT, 39 (23.6%) had positive test results.
**Hirko et al 2020 (** 35 **)**					
Colonoscopy, FIT, sigmoidoscopy	Pender’s Health Promotion Model; randomized controlled trial	CRC screening participation within 6 months after mailed letter	**Intervention group**: Receipt of motivational outreach letter with option to call and request FIT kit. Letter designed to address screening barriers in rural populations that included call to action that emphasized limited supply of FIT kits. **Control group**: Usual care mailed reminder letter.	A 7.6 percentage-point increase in screening participation in the intervention group (30.1%) vs control group (22.5%) (*P* < .001). Intervention vs control group had 49% higher odds of being screened over follow-up (OR, 1.49; 95% CI, 1.34–1.65).	No significant interaction between study group and sex (*P* = .54) or study group and BMI category (*P* = .39). No significant differences in FIT kit request, returns, or follow-up testing by sex in intervention group. FIT kit requests and follow-up testing were similar by BMI category; patients with obesity (57.3%) vs overweight (81.3%) and normal (67.4%) BMI less likely to return FIT kits (*P* = .01). Of patients who returned FIT, 18 (11.7%) had positive test results.
**Honeycutt et al 2013 (** 36 **)**					
Colonoscopy	None reported; quasi-experimental	Colonoscopy referral, examination, and guideline-compliant CRC screening at end of study based on abstracted medical record data	**Intervention clinics**: Integrated Community Cancer Screening Program into clinic. Program included multiple strategies to reduce barriers to colonoscopy, including patient health navigation and colonoscopy at reduced or no cost to patients. **Comparison clinics**: Clinics that did not have Integrated Community Cancer Screening Program.	Significantly higher percentage of colonoscopy referrals in intervention clinics (58%) vs comparison clinics (24%) (*P* < .001). Patients at intervention vs those at comparison clinics were significantly more likely to receive colonoscopy (35% vs 7%, *P* < .01) and be guideline-compliant (43% vs 11%, *P* < .001). Results remained significant in multilevel analysis adjusted for age and race.	NA
**Hountz et al 2017 (** 37 **)**					
Any test (colonoscopy or FIT)	Plan-Do-Study-Act; preintervention–Postintervention	CRC screenings ordered and completed	Six quality improvement interventions implemented: 1) protocol and algorithm for CRC screening; 2) simplified FIT ordering process; 3) clinical decision support tools; 4) letters sent to patients with outstanding FIT; 5) educational brochure on colonoscopy and FIT placed in patient rooms; 6) comments box in electronic health record within CRC screening windows.	Significant increase in number of screenings ordered (38% preintervention to 75% postintervention) and screenings completed (30% preintervention to 58% postintervention) (*P* values <.001).	Nineteen patients had positive test results from FIT or colonoscopy.
**Katz et al 2024 (** 38 **)**					
Colonoscopy, FIT	Protection Motivation Theory; pilot randomized controlled trial	Return of FIT kit at 2 months postmailing	**Video brochure intervention group**: Patients sent video brochure on CRC screening and FIT kit. Mailed reminder letter to complete FIT if not returned in 2 weeks. **Audio brochure intervention group**: Sent audio-recorded information on CRC screening and FIT kit. Mailed reminder letter to complete FIT if not returned in 2 weeks. **Usual care control group**: Sent FIT kit with manufacturer’s instructions.	Significantly higher FIT return rates in video brochure group (28%) vs audio brochure group (10%) (*P* = .045). FIT return rates in audio brochure group and video brochure group not different from usual care group (15%) (*P* = .53 and *P* = .28, respectively). FIT return rates higher among patients sent video brochure (*P* = .046).	Of patients who returned FIT, 2 (12.5%) had positive test results.
**Kinney et al 2014 (** 39 **)**					
Colonoscopy	Extended Parallel Process Model; randomized controlled trial	Medically verified colonoscopy within 9 months of intervention	**TeleCARE intervention group**: Mailed educational brochure and tailored visual aids, telephone session with cancer risk specialist using risk communication, behavioral change, motivational interviewing techniques. **Comparison group**: Mailed educational brochure based on familial risk status.	Significantly higher receipt of colonoscopy in TeleCARE group (34.5%) vs comparison group (15.7%) (*P* < .001).	Intent-to-treat analysis from imputed data show comparable intervention effect in rural areas (OR, 2.89; 95% CI, 1.53–5.46) and urban areas (OR, 2.87; 95% CI, 1.85–4.46). Intervention effect also similar by household income. The estimated direct cost of TeleCARE was $42.40 per participant and $8.20 for the print-only intervention (comparison group). Estimated total cost of TeleCARE intervention was $9,790, which was $287.95 for each additional colonoscopy received in this group.
**Klugas et al 2024 (** 40 **)**					
Any test (Cologuard, colonoscopy, FIT, gFOBT, sigmoidoscopy, or virtual colonoscopy)	None reported; quasi-experimental	Cologuard tests ordered and completed; overall CRC screening rates	Two brief online academic detailing sessions (offering continuing medical education credit [nonmonetary incentive]) with providers. Sessions included review of CRC screening guidelines, how to access and identify patients overdue for screening from electronic medical records, and how to order and track test completion and results. Providers were encouraged to spend 1 hour per week on panel management for CRC screening.	From preintervention to postintervention, significant increase in monthly screenings ordered (*P* < .01) and completed (*P* = .02), and weekly screenings completed (*P* < .01). Overall CRC screening rate increased significantly after intervention (69.7%) vs before intervention (64.3%) (*P* < .01).	NA
**Kluhsman et al 2012 (** 41 **)**					
FIT	Cognitive-social health information processing model; prospective, single-group, multiple-site pilot	Completed FIT	Receipt of take-home FIT kit and brochure during health care examination. Patients who did not complete FIT were referred for telephone counseling sessions using problem-solving techniques to address barriers to CRC screening. Booster calls were made to patients who remained unscreened to reinforce the importance of screening. “Cue to action” reminder message left after failed contact.	Overall FIT completion was 84%. Initial FIT completion was 72.5%, and 27.5% were referred for telephone counseling. Of those referred, 41.8% completed FIT after counseling. Among those referred for counseling, no significant difference in FIT completion between those who received counseling vs answering machine reminder.	No significant differences in FIT completion by sociodemographic characteristics examined (sex, age, education, marital status, income, insurance coverage, insurance coverage for CRC screening). Barriers significantly associated with odds of not completing FIT included benefits do not outweigh risks; normal test result does not lessen worry about CRC; early detection does not make CRC easier to treat; screening might be uncomfortable; and screening test too difficult to understand.
**Krok–Schoen et al 2015 (** 42 **)**					
Any test (colonoscopy, FOBT, or sigmoidoscopy)	Attitude Accessibility Theory, community-based participatory research; Health Belief Model, Social Cognitive Theory of Reasoned Action; randomized controlled trial	Completed CRC screening within USPSTF guidelines	**Intervention counties**: Media campaign (Waves 2 and 4) that featured local residents and included information about CRC risk factors, symptoms, and screening. Clinic intervention (Waves 3 and 4) included educational posters and brochures on CRC mortality rates and motivational messages. **Comparison counties**: Media campaign and patient education material related to healthy eating.	After adjustment for baseline screening, no significant difference in Wave 4 screening rates among intervention counties (35.2%) vs comparison counties (31.4%) (*P* = .50).	NA
**Levy et al 2012 (** 43 **)^h^**					
FIT	None reported; randomized controlled trial	Return of FIT	**Mailed education intervention group**: Patients received educational packet with information on CRC screening, DVD and booklet, FIT, and magnet. **Mailed education plus telephone reminder intervention group**: Educational intervention plus telephone call made by study team to educate, remind, and provide supportive feedback about CRC screening.	No significant difference in return of FIT between mailed education group (45.2%) vs mailed education plus telephone reminder group (48.7%) (*P* = .50). Significant increases observed in CRC screening when comparing rates to Medicare beneficiaries (*P* < .001).	Older age was significantly associated with higher FIT return rate (55.7% aged ≥65 years vs 43.5% aged <65 years; *P* = .03). Significant increase in attitudes toward and readiness for screening at follow-up (*P* values <.001). Barriers to testing were significantly lower after education intervention (*P* = .03). No significant differences in FIT return rates by marital status, income, or medical insurance status.
**Levy et al 2013 (** 44 **)^h^**					
Barium enema, colonoscopy, FIT, FOBT, sigmoidoscopy	None reported; randomized controlled trial	Completion of any CRC screening	**Chart reminder group**: Physicians received chart reminder for CRC screening. **Mailed education intervention group**: Chart reminder plus patients received educational packet with information on CRC screening, DVD and booklet, FIT, and magnet. **Mailed education plus telephone reminder intervention group**: Chart reminder and mailed education plus telephone call made by study team to educate, remind, and provide supportive feedback about CRC screening. **Usual care control group** : Patients received usual care.	CRC screening completion was 17.8% in usual care group, 20.5% in chart reminder group, 56.5% in mailed education group, and 57.2% in mailed education plus telephone reminder group. Compared with usual care, odds of CRC completion was higher for mailed education group (OR, 6.0; 95% CI, 3.7–9.6) and mailed education plus telephone reminder group (OR, 6.2; 95% CI, 3.8–9.9). No difference in odds between chart reminder and usual care groups (OR, 1.2; 95% CI, 0.7–2.0).	In adjusted analysis, other variables significantly associated with CRC screening included doctor recommendation of CRC (OR, 1.7; 95% CI, 1.2–2.4), higher perceived importance of CRC screening (OR, 1.8; 95% CI, 1.2–2.5), and having physical examination during follow-up period (OR, 1.5; 95% CI, 1.03–2.1).
**Moralez et al 2012 (** 45 **)**					
Colonoscopy, FOBT, sigmoidoscopy	None reported; preintervention–postntervention	CRC screening awareness and behavior	Community members hosted *promotora*-led “home health parties”; participants were taught about CRC screening via interactive presentation and discussion. Hosts were recruited from the community at outreach events. Participants received resource guide on CRC screening locations and, if interested, scheduling assistance from *promotoras*.	From baseline to follow-up, significant increase in awareness of FOBT (48.3% to 75.0%, *P* < .001) and colonoscopy or sigmoidoscopy (58.3% to 86.7%, *P* < .001). There was also a significant increase in the proportion who reported ever having a FOBT (31.1% to 41.0%, *P* = .014) and colonoscopy or sigmoidoscopy (29.5% vs 39.4%, *P* = .014).	NA
**Moss et al 2024 (** 46 **)**					
FIT	None reported; pilot randomized controlled trial	CRC screening uptake^i^	**Self-sampling intervention group**: Participants were mailed a package containing educational flyers, self-sampling tools with instructions written for participants with low literacy, and FIT. Reminder letters sent out to patients who did not return the FIT. **Standard-of-care control group**: Received letter to encourage scheduling an appointment for CRC screening.	CRC screening was 75% in the self-sampling group and 13% in the standard-of-care group (OR, 31.32, 95% CI, 5.20–289.33).	Knowledge of cancer screening at baseline was significantly associated with CRC screening (OR, 2.38; 95% CI, 1.10–5.90). No significant associations observed between health care trust and cancer fatalism and CRC screening.
**Preston et al 2018 (** 47 **)**					
FOBT	Community-based participatory research, Health Behavior Theory; randomized controlled trial	Return rate of FOBT screening kit within 60 days	**Tailored CRC risk intervention group**: Community lay health worker provided community-centered CRC education and demonstrated use of FOBT. Local role model presented experience with CRC. Participants received FOBT kit and brochure. **General CRC risk intervention group**: Academic health professional provided general CRC education. Participants received FOBT kit and brochure. **Cardiovascular disease risk control group**: Academic health professional provided education on cardiovascular disease risk and monitoring. Participants received FOBT and brochure about cardiovascular disease.	Overall FOBT return rate was 32%. Return rate was significantly higher in general CRC risk group (42%) vs tailored CRC risk group (28%; *P* = .04) and cardiovascular disease risk control group (25%; *P* = .0099).	NA
**Rawl et al 2023 (** 48 **)^j^**					
Colonoscopy, FIT or FOBT	None reported; qualitative formative evaluation of randomized controlled trial	Receipt, uptake, and satisfaction with CRC screening intervention content^k^	See Vachon et al 2024 (52) for intervention description.	Of intervention participants who completed a telephone interview for the formative evaluation, 76.9% viewed DVD content on CRC. Most (91.0%) strongly agreed or agreed that the DVD provided needed information to get screened for CRC, and 62.5% reported it helped them decide to get screened. CRC was discussed with patient navigators by 86.8% of participants. Lack of knowledge and screening not a priority were most commonly cited barriers.	NA
**Redwood et al 2012 (** 49 **)**					
Any test (colonoscopy, FOBT, or sigmoidoscopy)	None reported; 3 statewide pilot projects (varied designs)	CRC screening rates	Pilot projects included a sigmoidoscopy training program for nurse practitioners and physician assistants; provision of itinerant endoscopy services at rural tribal health facilities; creation and use of first-degree relative database to identify and screen people at increased risk for CRC; and patient navigation services to support and implement CRC screening.	The overall CRC screening rates in the Alaska Native Tribal Health System increased among Alaska Native patients from 41% before initiation of the projects to 55% in 2010.	NA
**Schlauderaff et al 2017 (** 50 **)**					
Any test (colonoscopy or FIT)	Plan-Do-Study-Act; preintervention–postintervention	Documented CRC screening in electronic medical records	Implementation of workflow changes and training for medical assistants. Workflow changes included but were not limited to development of protocols for medical assistants to order CRC screening; reminder systems; load leveling of staff; referral tracking systems; mass mail FIT to patients needing CRC screening; testing done at clinics where patients were eligible for sliding fee schedule and uncompensated care programs.	Improvement in documented CRC rates were achieved. The base rate of documented CRC screening was 22% vs 62.7% 2 years later.	NA
**Schlichting et al 2014 (** 51 **)**					
Barium enema, colonoscopy, FIT, FOBT	None reported; quasi-experimental	Returned FIT kit	**Low-intensity intervention group**: Patients were mailed a packet containing CRC screening educational materials and FIT kit with instructions. **High-intensity intervention group**: Same as low-intensity group plus incentive and reminder telephone calls.	Among eligible survey participants, FIT return rate was higher in the low-intensity group (92%) vs high-intensity group (45%) (*P* < .001). However, a higher proportion of FITs were returned of those mailed in the high-intensity group (85%) vs low-intensity group (14%).	The total cost per FIT returned was $44.86 in the high-intensity group and $27.43 in the low-intensity group. The total cost per CRC screen was $30.92 in the low-intensity group and $14.06 in the high-intensity group. Of patients who returned FIT, 18 (11%) had positive test results.
**Vachon et al 2024 (** 52 **)^j^**					
Colonoscopy, FIT, or FOBT	Health Belief Model, Transtheoretical Model; randomized controlled trial	Completed CRC screening within 12 months from randomization	**DVD-only intervention group**: Participants were mailed a tailored, interactive DVD that addressed factors influencing uptake of cancer screening. The DVD contained information about multiple cancer screenings; participants could opt to learn more about CRC screening if interested. **DVD plus patient navigation intervention group**: Same as DVD group plus patient navigation telephone sessions to encourage and support CRC screening. **Usual care control group**: Participants received health-related newsletters that did not include cancer information and were sent brochures on cancer screening at end of intervention.^l^	CRC screening completion was 26%. By group, 18% completed screening in the DVD-only group, 39% in the DVD plus patient navigation group, and 18% in the usual care group (*P* < .001). In adjusted model, DVD plus patient navigation group had significantly higher odds of completing CRC screening vs DVD-only group (OR, 3.86; 95% CI, 2.43–6.13; *P* < .001) and usual care group (OR, 3.62; 95% CI, 2.09–6.47; *P* < .001). No significant difference in adjusted odds between DVD and usual care groups (OR, 0.94; 95% CI, 0.52–1.72).	Perceived self-efficacy for CRC screening (*P* = .007) and intention to have colonoscopy in next 6 months (*P* < .001) significantly associated with CRC screening completion. Rural–urban commuting area and age not significantly associated with CRC screening.
**Westfall et al 2013 (** 53 **)**					
Barium enema, colonoscopy, FOBT, sigmoidoscopy, virtual colonoscopy	Community-based participatory research; quasi-experimental	CRC screening uptake	**Intervention region**: Awareness and educational campaign that encouraged eligible residents to discuss CRC screening with a physician. Messages addressed catalysts of behavior change and communicated via newsletters, small media, and organizations. **Control region**: No awareness and educational campaign.	Intent-to-treat analysis showed a 5 percentage-point absolute increase in the proportion of respondents who ever had any CRC screening in the intervention region (from 76% to 81%) vs no increase in the control region (77% at both time points) (*P* = .22). No significant differences in screening observed by individual CRC tests. Greater exposure to intervention materials was associated with significant increase in any CRC screening. In exposure analysis of follow-up data, higher intervention exposure was associated with greater knowledge of CRC and higher screening rates.	NA
**Woodall and DeLetter 2018 (** 54 **)**					
FIT	Human Caring Theory, Transtheoretical Model (Stages of Change); preintervention–postintervention	CRC knowledge, FIT kit returns	Brief (10 min) CRC education session and receipt of FIT kit.	Significant increase in mean CRC knowledge scores from preintervention (8.29) to postintervention (13.27) (range of scores: 0 to 14, higher scores indicating greater knowledge) (*P* < .001). Of 130 (70%) participants who elected to take a FIT kit home, 29 (15%) returned the kit.	Of participants who returned FIT during the study period, 5 (41.7%) had a positive test result.
**Zoellner et al 2023 (** 55 **)**					
Colonoscopy, FIT	Consolidated Framework for Implementation Research, Plan-Do-Study-Act; mixed methods (quasi-experimental and qualitative interviews)	FIT return rates	**Automated reminder group**: In first 2 weeks, automated electronic telephone reminders were sent per patient preference via text message, telephone, email, or electronic medical record portal. Third and last reminder delivered as live call by care coordinator. **Usual care group**: Live telephone reminders delivered by care coordinator. **Interviews**: Conducted among staff to explore how they recommended CRC screening to patients, barriers and facilitators to CRC screening, and perceptions of organizational change process related to the project.	Overall return rate in usual group (79%) was similar to automated reminder group (72%) (*P* = .24). Example barriers to completing CRC screening tests described in staff interviews included limited time and access, incorrect completion of FIT, and financial costs. Facilitators included supportive clinic processes, motivated organizational culture, and patient education.	Of patients who returned FIT, 4 (9.6%) had a positive test result.

Abbreviations: BMI, body mass index; FIT, fecal immunochemical test; FOBT, fecal occult blood test; gFOBT, guaiac fecal occult blood test; NA, not applicable; USPSTF, US Preventive Services Task Force.

a Tests that were delivered or evaluated as a CRC screening outcome (eg, percentage screened). Includes tests performed as initial screening and diagnostic follow-up.

b Theories, frameworks, or research approaches (eg, community-based participatory research) used to inform the development, implementation, or evaluation of the intervention.

c Describes main or relevant findings pertaining to CRC screening, sociodemographic or health-related differences in CRC screening, and evaluations of cost effectiveness. The results reported in this table are not meant to serve as an exhaustive list (ie, NA does not mean that no additional results were reported). Refer to each study for a detailed description of findings.

d Arnold et al 2016 and Davis et al 2013 report on the same intervention. Davis et al 2013 report on return of initial FOBT within 3 months and 12 months (initial results), while Arnold et al 2016 report return of 1, 2, or 3 FOBTs over a 3-year period (final results).

e Arnold et al 2019 and Davis et al 2020 report on the same intervention. Arnold et al 2019 report on return of initial FIT within 12 months, while Davis et al 2020 report on return of repeat FIT within 12 to 18 months.

f Listed as 51.0% in the referenced article’s abstract.

g Listed as $134.61 in the referenced article’s abstract.

h Levy et al 2012 and Levy et al 2013 report on the same intervention. Levy et al 2012 report results for all 4 study groups (chart reminder group, mailed education group, mailed education and telephone reminder group, usual care control group), while Levy et al 2013 compare results between 2 study groups (mailed education group vs mailed education and telephone reminder group).

i Intervention also included materials for and evaluation of cervical cancer screening; for the purposes of this review, we report only on CRC screening.

j Rawl et al 2023 and Vachon et al 2024 report results from the same intervention. Rawl et al 2023 report data on receipt, uptake, and satisfaction of the intervention, while Vachon et al 2024 report on CRC screening outcomes.

k The information reported here describes results for CRC screening only. Results on other cancer screening content included in the original intervention are reported in the article cited.

l Description of usual care group, as well as detailed description of intervention activities, are reported in the baseline publication: Biederman E, Baltic R, Katz ML, Rawl S, Vachon E, Monahan PO, et al. Increasing breast, cervical, and colorectal cancer screening among rural women: baseline characteristics of a randomized control trial. *Contemp Clin Trials*. 2022;123:106986. doi:10.1016/j.cct.2022.106986

### Intervention strategies and approaches

Interventions used various approaches to increase CRC screening completion or follow-up (Table 3). Of the 36 studies, 34 were multicomponent interventions. Of these studies, 27 implemented approaches from 2 or more of the 3 CPSTF-recommended strategies: increase community demand, increase community access, and increase provider delivery of screening services.

**Table 3:** Intervention Strategies and Approaches^a^ of Included Studies (N = 36), Scoping Review of Colorectal Cancer Screening Interventions in Rural Settings, January 2010–May 2024

Study	Increase community demand						Increase community access		Increase provider delivery of screening services			Total
	Client reminders	Client incentives	Small media	Mass media	Group education	One-on-one education	Reduce structural barriers	Reduce out-of-client pocket costs	Provider assessment and feedback	Provider incentives	Provider reminders	
Adegboyega et al 2022 (20)						●	●					2
Arnold et al 2016 (21)^b^	●		●			●	●					4
Arnold et al 2019 (22)^c^	●		●			●	●					4
Briant et al 2015 (23)	●		●		●	●	●	●				6
Briant et al 2018 (24)			●		●		●					3
Charlton et al 2014 (25)			●				●					2
Christiansen et al 2016 (26)									●			1
Conn et al 2020 (27)	●								●			2
Curry et al 2011 (28)									●	●		2
Davis et al 2013 (29)^c^	●		●			●	●					4
Davis et al 2020 (30)^d^	●		●			●	●					4
Davis et al 2023 (31)	●						●					2
Dignan et al 2014 (32)										●		1
Hardin et al 2020 (33)	●	●				●	●					4
Haverkamp et al 2020 (34)	●					●	●					3
Hirko et al 2020 (35)			●				●					2
Honeycutt et al 2013 (36)	●					●	●	●	●		●	6
Hountz et al 2017 (37)			●						●		●	3
Katz et al 2024 (38)	●		●				●					3
Kinney et al 2014 (39)	●		●			●						3
Klugas et al 2024 (40)	●		●			●	●		●	●		6
Kluhsman et al 2012 (41)	●		●			●	●					4
Krok–Schoen et al 2015 (42)	●		●			●						3
Levy et al 2012 (43)^d^	●		●			●						3
Levy et al 2013 (44)^d^	●		●			●					●	4
Moralez et al 2012 (45)			●		●		●					3
Moss et al 2024 (46)	●		●				●					3
Preston et al 2018 (47)	●		●		●		●					4
Rawl et al 2023 (48)^e^	●		●			●	●					4
Redwood et al 2012 (49)	●					●	●		●	●		5
Schlauderaff et al 2017 (50)	●						●	●			●	4
Schlichting et al 2014 (51)	●		●				●					3
Vachon et al 2024 (52)^e^			●			●	●					3
Westfall et al 2013 (53)			●	●								2
Woodall and DeLetter 2018 (54)	●		●			●						3
Zoellner et al 2023 (55)	●		●			●						3
**Total**	25	1	25	1	4	20	24	3	7	4	4	

a Categorized according to Community Preventive Services Task Force–recommended strategies for multicomponent interventions for colorectal cancer screening.

b Arnold et al 2016 and Davis et al 2013 report on the same intervention. Davis et al 2013 report on return of initial FOBT within 3 months and 12 months (initial results), while Arnold et al 2016 report return of 1, 2, or 3 FOBTs over a three-year period (final results).

c Arnold et al 2019 and Davis et al 2020 report on the same intervention. Arnold et al 2019 report on return of initial FIT within 12 months, while Davis et al 2020 report on return of repeat FIT within 12 to 18 months.

d Levy et al 2012 and Levy et al 2013 report on the same intervention. Levy et al 2012 report results for all 4 study groups (chart reminder group, mailed education group, mailed education and telephone reminder group, usual care control group), while Levy et al 2013 compare results between 2 study groups (mailed education group vs mailed education and telephone reminder group).

e Rawl et al 2023 and Vachon et al 2024 report results from the same intervention. Rawl et al 2023 report data on receipt, uptake, and satisfaction of the intervention, while Vachon et al 2024 report on CRC screening outcomes.

#### Increase community demand

The most common approaches used to increase community demand for screening were client reminders (n = 25), small media (n = 25), and one-on-one education (n = 20). Client reminders were sent to people due for CRC screening or follow-up and were typically done via telephone calls and mailed letters from health care staff. Small media approaches included educational pamphlets, visual aids, brochures, short videos, and audio-recorded information about CRC screening. Some small media was either culturally tailored or provided tailored messaging based on a person’s family history or health beliefs. Fewer studies used group education (n = 4), mass media (n = 1), or client incentives to encourage screening (n = 1).

#### Increase community access

Twenty-four studies focused on reducing structural barriers to CRC screening (Table 3). Most commonly, interventions reduced barriers to accessing and receiving CRC screening by mailing FIT kits with prestamped, preaddressed return envelopes. Some interventions used a lay health advisor or patient navigator approach; for example, Vachon et al 2024 (52) used patient navigators to help with screening, including arranging transportation for interested patients. Three studies addressed cost barriers by offering — or partnering with organizations to provide — reduced-cost colonoscopies for screening or diagnostic testing. For example, Briant et al 2015 (23) worked with local hospitals, clinics, and programs to provide free or low-cost colonoscopies to participants with a positive FOBT test result.

#### Increase provider delivery of screening services

Ten studies implemented approaches to increase provider delivery of screening services (Table 3). Of these studies, 7 used provider assessment and feedback, 4 implemented provider reminders, and 4 used provider incentives to increase CRC screening. Provider incentives were largely confined to academic detailing, which can offer continuing medical education credit (nonmonetary incentive), to enhance provider knowledge and delivery of CRC screening. Multiple studies used quality improvement methods to facilitate CRC screening, often combining 1 or more approaches as part of this strategy. For example, Schlauderaff et al (50) used the Plan-Do-Study-Act model to inform implementation of multiple workflow processes, including a provider reminder system to assist with follow-up testing.

### Intervention outcomes and results

Most studies observed an increase in CRC screening following the intervention. Our results, including examples from selected studies, are summarized below.

#### CRC screening uptake

Multiple studies reported a significant increase in CRC screening compared with usual care or control conditions. Charlton et al 2014 (25) observed significantly higher CRC screening (21%) with a mailed education and FIT kit intervention compared with both education only (6%) and usual care (6%) (*P* values < .001). Hardin et al 2020 (33) implemented a patient incentive and navigation program, which included patient reminders and assistance with screening barriers. The FIT return rate increased from 21.7% during the usual care period to 47.6% during the implementation period (*P* = .001).

Haverkamp et al 2020 (34) tested the effectiveness of 2 interventions for American Indian or Alaska Native patients: 1) mailing FIT kits alone and 2) mailing plus telephone or home outreach from an American Indian Community Health Representative. The percentage of completed FIT kits was significantly higher in both intervention groups (16.9% and 18.8%) compared with usual care (6.4%) (*P* values < .01). Hirko et al 2020 (35) tested the effectiveness of a mailed motivational message reminder for CRC screening and found a significant increase in CRC screening in the intervention group (30.1%) vs control group (22.5%) (*P* < .001).

Some studies examined intervention effectiveness without comparison to usual care. In 2 related studies, Arnold et al 2019 (22) and Davis et al 2020 (30) compared the relative effectiveness of a personal call vs automated call intervention. The percentage of patients who had ever completed a FOBT was similar at baseline in both personal call (20%) and automated call (17%) groups (22). While the study found no significant difference in return of initial FIT kit within 12 months between groups, the screening prevalence in both groups was nearly 70% postintervention (22). Uptake of repeat FIT within 12 to 18 months of initial FIT was also comparable in both groups (33.6% in personal call vs 36.5% in automated call group) (30).

Four studies did not observe significant improvements in CRC screening for their main outcome or in primary analysis (20,32,42,53). For example, Adegboyega et al 2022 (20) evaluated a motivational interviewing intervention provided by lay health advisors and observed no significant difference in CRC screening between the intervention and the control group (12% vs 15%; *P* = .72). Westfall et al 2013 (53) implemented a county-level awareness and education campaign that encouraged residents to get screened. They found no significant differences in reported CRC screening between intervention and control regions (81% vs 77%; *P* = .22), although the authors did report a significant increase in screening at higher levels of intervention exposure at follow-up.

Some studies examined CRC screening but did not report on significance or include a usual care or control group. For example, Christiansen et al 2016 (26) used patient empanelment (ie, assigned patients to a primary care provider and designated care team) and implemented systems-level and practice-level changes to promote CRC screening, which increased from 9% at baseline to 19% at 12 months postempanelment. Davis et al 2023 (31) conducted a mixed-methods, single-arm exploratory pilot study to evaluate the preliminary effectiveness of a mailed FIT and patient reminder intervention. Patients who were not up to date with CRC screening were eligible to participate. At 12 months postmailing, 21% of patients had completed a mailed FIT and 15% had completed a clinic-distributed FIT or other screening.

Multiple studies conducted subgroup analyses to examine differences in the effect of the intervention(s) on CRC screening by sociodemographic and health-related characteristics, with varied results. For example, Arnold et al 2019 (22) and Davis et al 2020 (30) found no significant differences in initial or repeat FIT completion between study groups among people with limited health literacy or adequate health literacy (*P* values range, .12 to .77). Davis et al 2020 (30) found treatment effects by age; the personal call vs automated call intervention was more effective among participants aged 60 years or older, while the automated call intervention was more effective among participants aged 50 to 59 years (*P* = .01). Charlton et al 2014 (25) and Hirko et al 2020 (35) did not find significant differences in screening prevalence by sex and study group.

#### Other outcomes

Some studies examined outcomes beyond screening uptake, including CRC screening familiarity, intent, and referral. Briant et al 2015 (23) held community fairs to provide education on and awareness of CRC screening. The authors reported a significant increase in familiarity with CRC screening among numerous demographic groups as well as associations between older age and Hispanic ethnicity, with higher odds of CRC screening intent after intervention. In a subsequent study, Briant et al 2018 (24) implemented *promotor(a)*-led “home health parties” and reported an increase in CRC screening awareness and knowledge, as did Woodall and DeLetter 2018 (54), who implemented brief CRC education sessions. Honeycutt et al 2013 (36) abstracted medical record data to evaluate an integrated community cancer screening program for CRC implemented between 2009 and 2011. The authors found a significantly higher percentage of colonoscopy referrals in intervention clinics (58.0%) compared with comparison clinics (23.9%) (*P* < .001).

#### Intervention costs

Six studies conducted analyses to examine costs associated with the intervention, which varied with scope and scale of the intervention used. For example, Christiansen et al 2016 (26) reported a total project cost of $52,987 to assign a designated primary care provider for patients, along with other systems-level and practice-level changes. Conn et al 2020 (27) reported the average total cost to track patients and mail reminders per FIT test returned as $60.18. The average cost of reminders only per FIT test returned was $11.20. Davis et al 2013 (29) reported incremental costs of $250 per additional person screened over usual care for the educational intervention and $1,337 for the nurse support intervention.

## Discussion

The purpose of this scoping review was to describe the published literature on interventions to increase CRC screening in rural settings. The interventions included in our review were largely effective at increasing CRC screening uptake. The most common intervention approaches were client reminders (n = 25), small media (n = 25), and reducing structural barriers (n = 24). Several studies focused on reducing structural barriers used patient navigation. In 2022, the CPSTF recommended patient navigation based on strong evidence of its effectiveness in increasing CRC screening and reducing inequities (58). Patient navigation is also a cost-effective strategy (58) — an important consideration in rural settings with fewer resources for intervention implementation.

Thirty-four studies implemented multicomponent interventions, and most of these studies combined 2 or more of the 3 CPSTF-recommended intervention strategies. Evidence from CPSTF suggests that multicomponent interventions are more effective when they combine intervention strategies to increase community demand for and access to cancer screening (15). Sharma et al 2022 (59) further demonstrated the benefit of multicomponent interventions among clinics participating in a national CRC screening program. The largest increases in annual CRC screening rates were observed among clinics that implemented 3 or more evidence-based interventions (59).

Our review shows that use of individual-level approaches (eg, one-on-one education) were more common than systems-level approaches (eg, quality improvement), though several studies implemented approaches at multiple levels. Multilevel interventions offer benefits over single-level interventions, including the ability to intervene upon multiple causes of health disparities and produce positive synergistic effects (60). Previous studies have identified barriers to CRC screening at multiple levels, including patient (eg, no health care coverage), provider (eg, lack of awareness of CRC screening protocols), systems (eg, shortage of specialists), and county (eg, high county-level rates of poverty), emphasizing the importance of implementing interventions that can reduce barriers at each level (61).

### Rural settings

Most studies did not provide a formal definition of rurality, limiting our understanding of the contexts in which these interventions might be most effective. The definition of rurality, as well as its measurement, has changed and become more complex over time (62). Depending on the definition of rural used, the population can include as much as 19% of the US population (59 million people) (63). None of the available rural classification schemes are considered a gold standard definition of rurality.

Shifting definitions of rurality challenge the ability of researchers and public health practitioners to review and compare data and findings, assess the suitability of interventions for their populations of interest, and determine similarities in community need and context. Future studies should be clear about how rurality is defined so that it is possible to determine potential relevance to one’s own context. Defining rurality also allows for better comparison of screening rates across different settings described as rural, including whether expected outcomes are achieved across diverse settings.

States with large Appalachian regions known to have lower CRC screening prevalence (eg, Kentucky, Ohio) (64) were well represented among the studies meeting our inclusion criteria. However, other states in the southern Appalachian region with large rural populations (eg, Tennessee, North Carolina, South Carolina) were not. The populations in these states include rural residents of varying racial and ethnic groups, including Black, White, Hispanic, and American Indian or Alaska Native. Distance and travel time to health care facilities are also important considerations for rural residents (65) and can vary widely by geographic region. As illustrated in Figure 2, we did not identify studies in several states with the highest CRC incidence, including southern states such as Mississippi and Alabama. This could indicate that populations in greatest need are not being evaluated in studies for the best approaches to effectively reach them.

The national CRC screening program evaluated in Sharma et al 2022 (59) provides an opportunity to improve evidence-based implementation of CRC screenings in rural settings. The program works with health care systems, academic institutions, and other organizations to strengthen intervention strategies for CRC screening (66). The focus on serving people with lower incomes and reaching across multiple states makes the program uniquely positioned to address barriers to screening in rural settings. The program currently operates with 35 funded recipients (66) and could be expanded to states where we identified no studies meeting our inclusion criteria.

### Costs

A small number of studies included data on intervention costs. Rural communities face unique economic and access challenges that can hinder intervention delivery, implementation, and participation. Less access to specialty care (eg, endoscopist), longer travel distances to facilities for in-person screening services, and greater financial burden due to higher uninsured rates are important considerations in rural settings (14,67). Understanding intervention costs can help communities decide which evidence-based interventions might be most feasible to implement. For example, mean costs are lower for FIT/FOBT-based programs than colonoscopy (68) and thus may be more feasible for initial screening of average-risk adults in rural health care systems. Multicomponent interventions that are delivered remotely can reduce access barriers and increase CRC screening rates (39).

### Race and ethnicity

Few studies provided a comprehensive examination of differences in the effect of CRC screening interventions by characteristics such as race and ethnicity. Black and American Indian or Alaskan Native people have the highest overall CRC incidence rates, with Black people experiencing the highest mortality (69). Accessible CRC screening could make a substantial impact on CRC burden in these groups, as shown by studies included in our review such as Redwood et al (49). Opportunities for future research include 1) conducting focused intervention recruitment among subgroups underserved by screening and 2) understanding how different intervention strategies might vary in effectiveness given the intersection of rurality and characteristics such as race and ethnicity.

### Strengths and limitations

One study strength is our use of broad search criteria that covered a 10-year span, allowing for greater inclusion of studies to inform our understanding of CRC intervention implementation and effectiveness. This study also has limitations. Not all studies used a randomized controlled trial design or included a control group, limiting understanding of how interventions might fare compared with usual care. Our review includes only peer-reviewed publications and does not account for interventions that have not been described in publications. Given changes in screening guidelines over time, some of the included studies delivered or evaluated tests that are no longer recommended (eg, barium enema).

### Conclusion

This scoping review of CRC screening is, to our knowledge, the first to focus primarily on rural settings. Most studies implemented multicomponent interventions and were effective at increasing CRC screening uptake. The most commonly implemented approaches were client reminders, small media, and reducing structural barriers. While most studies evaluated multicomponent interventions, we had insufficient data to evaluate which intervention approaches, or combinations of approaches, could have the greatest effect on CRC screening in rural settings. This effect is an important consideration for implementation, particularly in rural health care clinics that are often overstretched and understaffed (70). Understanding how and why an intervention is effective can demonstrate its feasibility and build evidence on what works in rural settings.

Opportunities for future research and practice include developing a uniform definition of rurality, using theory to inform intervention development, collecting data on intervention costs, and evaluating how intervention effectiveness might vary by intervention strategies and across subgroups. Expanded implementation of CRC screening efforts across the US — and particularly in states and regions with large rural populations — may help to ensure all residents can receive timely CRC screening.

## Appendix Search Strategy

**Table:** Medline (OVID) 1946–: Ovid MEDLINE(R) ALL <1946 to May 30, 2024>

Search number	Query	Results
1	exp colorectal neoplasms/	246,357
2	exp colonoscopy/	35,981
3	(colonoscopy or sigmoidoscopy or ((colorectal or fecal or stool or CRC) adj3 (test* or screen*)) or FOBT or "fecal occult blood test" or IFOBT).ti,ab.	51,672
4	((fecal* or feces) adj4 (immunochem* or immunohist*) adj4 test*).ti,ab.	1,667
5	exp Mass Screening/ and exp Colorectal Neoplasms/	8,370
6	2 or 3 or 4	67,088
7	1 and 6	28,320
8	5 or 7	29,959
9	(rural* or countryside* or backcountr* or backwater* or nonmetro* or non metro* or Appalachia* or regional health* or geographically isolat* or ((frontier* or remote* or isolated* or farming*) adj4 (county or counties or area* or communit* or neighborhood* or residence* or town* or village* or region* or province* or setting* or population*))).mp. or exp Rural Health Services/ or exp Rural Health/ or exp Rural Population/	26,5228
10	8 and 9	439
11	(intervention* or program* or strategy or strategies or uptake or implement* or increase or improve* or approach or evaluation* or validation* or clinical*).ti,ab. or exp Clinical Trial/ or Validation Study/ or Clinical Trials as Topic/ or Evaluation Study/	13,890,010
12	10 and 11	348
13	exp United States/ or ("United States" or USA or US or Alabama or Alaska or Arizona or Arkansas or California or Colorado or Connecticut or Delaware or Florida or Georgia or Hawaii or Idaho or Illinois or Indiana or Iowa or Kansas or Kentucky or Louisiana or Maine or Maryland or Massachusetts or Michigan or Minnesota or Mississippi or Missouri or Montana or Nebraska or Nevada or "New Hampshire" or "New Jersey" or "New Mexico" or "New York" or "North Carolina" or "North Dakota" or Ohio or Oklahoma or Oregon or Pennsylvania or "Rhode Island" or "South Carolina" or "South Dakota" or Tennessee or Texas or Utah or Vermont or Virginia or Washington or "West Virginia" or Wisconsin or Wyoming or "New England" or "Mid West*" or "West Coast" or "East Coast" or Appalachia* or "African American*" or "Asian American*" or "Native American*" or "American Indian*" or "Mexican American*" or "Southern Border").tw.	2,491,541
14	12 and 13	217
15	limit 14 to yr="2010 - 2024"	172

**Table:** Embase (OVID) 1974–: Embase <1988 to 2024 Week 22>

Search number	Query	Results
1	exp colorectal neoplasms/	458,620
2	exp colonoscopy/	107,931
3	(colonoscopy or sigmoidoscopy or ((colorectal or fecal or stool or CRC) adj3 (test* or screen*)) or FOBT or "fecal occult blood test" or IFOBT).ti,ab.	94,551
4	((fecal* or feces) adj4 (immunochem* or immunohist*) adj4 test*).ti,ab.	3,025
5	exp Mass Screening/ and exp Colorectal Neoplasms/	31,118
6	2 or 3 or 4	136,728
7	1 and 6	59,333
8	5 or 7	70,523
9	(rural* or countryside* or backcountr* or backwater* or nonmetro* or non metro* or Appalachia* or regional health* or geographically isolat* or ((frontier* or remote* or isolated* or farming*) adj4 (county or counties or area* or communit* or neighborhood* or residence* or town* or village* or region* or province* or setting* or population*))).mp. or exp Rural Health Services/ or exp Rural Health/ or exp Rural Population/	295,188
10	8 and 9	908
11	(intervention* or program* or strategy or strategies or uptake or implement* or increase or improve* or approach or evaluation* or validation* or clinical*).ti,ab. or exp "Clinical Trial (topic)"/ or Validation Study/ or Evaluation Study/	16,745,849
12	10 and 11	715
13	exp United States/ or ("United States" or USA or US or Alabama or Alaska or Arizona or Arkansas or California or Colorado or Connecticut or Delaware or Florida or Georgia or Hawaii or Idaho or Illinois or Indiana or Iowa or Kansas or Kentucky or Louisiana or Maine or Maryland or Massachusetts or Michigan or Minnesota or Mississippi or Missouri or Montana or Nebraska or Nevada or "New Hampshire" or "New Jersey" or "New Mexico" or "New York" or "North Carolina" or "North Dakota" or Ohio or Oklahoma or Oregon or Pennsylvania or "Rhode Island" or "South Carolina" or "South Dakota" or Tennessee or Texas or Utah or Vermont or Virginia or Washington or "West Virginia" or Wisconsin or Wyoming or "New England" or "Mid West*" or "West Coast" or "East Coast" or Appalachia* or "African American*" or "Asian American*" or "Native American*" or "American Indian*" or "Mexican American*" or "Southern Border").tw.	2,754,095
14	12 and 13	371
15	limit 14 to yr="2010 - 2024"	328

**Table:** CINAHL (EbscoHost)^a^

Number	Query
S10	S8 AND S9
S9	( (MH "Clinical Trials") AND (MH "Validation Studies") AND (MH "Evaluation Research") ) OR TI ( (intervention* or program* or strategy or strategies or uptake or implement* or increase or improve* or approach or evaluation* or validation* or clinical*) ) OR AB ( (intervention* or program* or strategy or strategies or uptake or implement* or increase or improve* or approach or evaluation* or validation* or clinical*) )
S8	S6 AND S7
S7	( (MH "Rural Health") AND (MH "Rural Health Services") AND (MH "Rural Health Centers") ) OR TI ( (rural* or countryside* or backcountr* or backwater* or nonmetro* or non metro* or Appalachia* or regional health* or geographically isolat* or ((frontier* or remote* or isolated* or farming*) N4 (county or counties or area* or communit* or neighborhood* or residence* or town* or village* or region* or province* or setting* or population*))) ) OR AB ( (rural* or countryside* or backcountr* or backwater or nonmetro* or non metro* or Appalachia* or regional health* or geographically isolat* or ((frontier* or remote* or isolated* or farming*) N4 (county or counties or area* or communit* or neighborhood* or residence* or town* or village* or region* or province* or setting* or population*)))
S6	S4 OR S5
S5	(MH "Health Screening") AND (MH "Colorectal Neoplasms")
S4	S2 OR S3
S3	TI ( ((fecal* or feces) N4 (immunochem* or immunohist*) N4 test*) ) OR AB ( ((fecal* or feces) N4 (immunochem* or immunohist*) N4 test*) )
S2	(MH "Colonoscopy") OR TI ( (colonoscopy or sigmoidoscopy or ((colorectal or fecal or stool or CRC) N3 (test* or screen*)) or FOBT or "fecal occult blood test" or IFOBT) ) OR AB ( (colonoscopy or sigmoidoscopy or ((colorectal or fecal or stool or CRC) N3 (test* or screen*)) or FOBT or "fecal occult blood test" or IFOBT) )
S1	(MH "Colorectal Neoplasms")

^a^ Limiters: Pub Year: 2010–2024; Geography: USA.

**Table:** Scopus

( ( TITLE-ABS ( intervention* OR program* OR strategy OR strategies OR uptake OR implement* OR increase OR improve* OR approach OR evaluation* OR validation* OR clinical* ) OR INDEXTERMS ( "evaluation study" ) OR INDEXTERMS ( "clinical trial" ) OR INDEXTERMS ( "validation study" ) OR INDEXTERMS ( "clinical trials as topic" ) ) AND ( ( ( INDEXTERMS ( "colorectal neoplasms" ) OR INDEXTERMS ( colonoscopy ) ) OR ( TITLE-ABS ( colonoscopy OR sigmoidoscopy OR ( ( colorectal OR fecal OR stool OR crc ) W/3 ( test* OR screen* ) ) OR fobt OR "fecal occult blood test" OR ifobt ) ) OR ( TITLE-ABS ( ( fecal* OR feces ) W/4 ( immunochem* OR immunohist* ) W/4 test* ) ) ) AND ( ( INDEXTERMS ( "rural health" ) OR INDEXTERMS ( "rural health services" ) ) OR ( TITLE-ABS-KEY ( rural* OR countryside* OR backcountr* OR backwater* OR nonmetro* OR "non metropolitan" OR appalachia* OR "regional health" OR "geographically isolated" OR ( ( frontier* OR remote* OR isolated* OR farming* ) W/4 ( county OR counties OR area* OR communit* OR neighborhood* OR residence* OR town* OR village* OR region* OR province* OR setting* OR population* ) ) ) ) ) ) ) AND TITLE-ABS-KEY ( "United States" OR usa OR us OR alabama OR alaska OR arizona OR arkansas OR california OR colorado OR connecticut OR delaware OR florida OR georgia OR hawaii OR idaho OR illinois OR indiana OR iowa OR kansas OR kentucky OR louisiana OR maine OR maryland OR massachusetts OR michigan OR minnesota OR mississippi OR missouri OR montana OR nebraska OR nevada OR "New Hampshire" OR "New Jersey" OR "New Mexico" OR "New York" OR "North Carolina" OR "North Dakota" OR ohio OR oklahoma OR oregon OR pennsylvania OR "Rhode Island" OR "South Carolina" OR "South Dakota" OR tennessee OR texas OR utah OR vermont OR virginia OR washington OR "West Virginia" OR wisconsin OR wyoming OR "New England" OR "Mid West*" OR "West Coast" OR "East Coast" OR appalachia* OR "African American*" OR "Asian American" OR "Native American" OR "American Indian" OR "Mexican American" OR "Southern Border" ) AND PUBYEAR > 2009 AND PUBYEAR < 2025
